# Gen Z Youth in the Battleground: Can AI Interventions Mitigate Risky Gaming Behaviours and Mental Health Harm?

**DOI:** 10.3390/ejihpe16050067

**Published:** 2026-05-12

**Authors:** Mostafa Aboulnour Salem

**Affiliations:** Deanship of Development and Quality Assurance, King Faisal University, Al-Ahsa 31982, Saudi Arabia; masalem@kfu.edu.sa

**Keywords:** AI cyber-shielding, anxiety symptoms, depressive symptoms, excessive gaming, Gen Z, hostile behaviours, personal well-being, PLS-SEM, prevention, risky behaviours, Sustainable Development Goals (SDGs)

## Abstract

Excessive gaming (EG) is increasingly recognised as a modifiable behavioural risk among youth, with potential implications for mental health and well-being in digitally mediated environments. This study examines excessive gaming as a behavioural exposure and AI cyber-shielding (AI-CS) as a perceived digital protective factor among Generation Z university gamers. AI-CS is conceptualised as users’ perceived exposure to AI-enabled safety mechanisms embedded in gaming-related digital environments. The study investigates the associations of EG and AI-CS with four psychological outcomes: depressive symptoms (DEP), anxiety symptoms (ANX), hostile behaviours (HB), and personal well-being (PWB). Data were collected through a cross-sectional online survey of 983 university students aged 18 to 22 years from multicultural Middle Eastern backgrounds enrolled in Saudi universities. The sample included 54.2% males and 45.8% females. The proposed relationships were analysed using Partial Least Squares Structural Equation Modelling (PLS-SEM). The results show that excessive gaming is positively associated with depressive and anxiety symptoms and negatively associated with personal well-being. No significant association was found between excessive gaming and hostile behaviours. AI cyber-shielding is negatively associated with depressive and anxiety symptoms and positively associated with personal well-being, but it does not significantly predict hostile behaviours. These findings indicate that excessive gaming is primarily associated with internal psychological outcomes rather than external behavioural responses. They also suggest that perceived AI-enabled safety affordances in gaming-related digital environments are associated with lower psychological distress and higher well-being. The study contributes to research on digital well-being by introducing and empirically examining AI cyber-shielding as a perception-based environmental factor associated with psychological functioning among university students.

## 1. Introduction

Individuals born between 1997 and 2012 are known as Generation Z. This group is the first to grow up fully immersed in digital technology. They are often called “digital natives” because online communication, social media, and digital interaction are part of their daily lives (Yordudom et al., 2026). Digital gaming is one of their main activities. It is not only for entertainment. It is also a space for communication, collaboration, and real-time interaction (Douglas, 2016).

The growth of digital gaming has attracted attention from public health and mental health research. The World Health Organisation recognised gaming disorder as a behavioural condition in the ICD-11 (World Health Organization, 2019). It is defined by poor control over gaming and continued use despite negative outcomes. The DSM-5-TR also identifies Internet Gaming Disorder as a condition that needs further study (Fam, 2018).

Most individuals play games without serious problems. However, a small proportion develop problematic gaming behaviour. A global review shows that gaming disorder affects about 1.2% to 5.9% of the population (Stevens et al., 2021). Earlier studies also show that 2–3% of gamers may meet clinical criteria. A larger group experiences mild psychological strain due to excessive gaming (Fam, 2018). Research also shows that excessive gaming is linked to depression, anxiety, sleep problems, and lower well-being (Salem & Khalil, 2026).

In this study, excessive gaming (EG) is defined as persistent or poorly controlled gaming that interferes with daily life or well-being. It is treated as a behavioural risk exposure rather than a clinical disorder (Daniels et al., 2026). This approach follows public health models that view risky behaviours as modifiable factors linked to negative outcomes.

Previous studies have focused mainly on behaviour. They examine gaming time and intensity as key factors. However, results are not consistent. This is especially true for external behaviours such as hostility. This suggests that behaviour alone cannot explain all psychological outcomes.

At the same time, digital environments are changing. Many platforms now use artificial intelligence (AI). AI systems include automated moderation, toxicity detection, and safety tools (Salem & Khalil, 2026). These systems affect what users see and how they interact. They work through visible features such as filtering, warnings, muting, and behaviour flags. Studies show that these systems influence user behaviour and perceived safety (Phillips, 2025).

In this study, AI cyber-shielding (AI-CS) is defined as a perception-based construct. It reflects how users experience and evaluate AI-based safety features in gaming environments. These features include moderation, toxicity detection, behaviour monitoring, and safety prompts (Lukings et al., 2026). AI-CS does not refer to one system. It represents users’ overall experience with different AI safety tools.

AI cyber-shielding is based on real systems used in gaming platforms. Examples include moderation tools in Riot Games, Xbox Safety Toolkit, Activision ToxMod, Discord, and Steam (Lukings et al., 2026). These systems detect harmful behaviour and respond with warnings, filtering, or intervention. Users interact with these systems through visible features rather than through technical knowledge of algorithms.

Despite this progress, most research still focuses on user behaviour. Digital environments are often treated as passive. Few studies examine how platform-level AI systems affect mental health. As a result, the role of AI-based environmental protection is still unclear and underexplored (Salem, 2026c).

This study addresses this gap. It introduces AI cyber-shielding as a measurable construct. It also distinguishes between two key dimensions: behavioural risk (excessive gaming) and environmental protection (AI cyber-shielding). This approach moves beyond behaviour-only models. It shows that both user actions and platform conditions influence psychological outcomes.

In this framework, excessive gaming represents risk exposure. AI cyber-shielding represents a protective environmental condition. The two constructs are treated as independent predictors. This approach follows protective factor models, in which risk and protection operate independently (Almuhtadi & Alageel, 2025).

This study examines the relationships between excessive gaming, AI cyber-shielding, and four outcomes: depression, anxiety, hostile behaviour, and well-being. The study uses a cross-sectional survey and PLS-SEM analysis. It provides a unified model to understand how behaviour and digital environments shape mental health. To improve clarity, the research objectives and questions are presented in Table 1.

## 2. Literature Review and Hypotheses

### 2.1. Excessive Gaming as a Risky Behaviour

Excessive gaming is increasingly recognised as a behavioural risk factor associated with adverse mental health outcomes, particularly depressive and anxiety symptoms. Prior research suggests that prolonged or uncontrolled gaming may function as a maladaptive coping strategy, allowing individuals to escape academic stress, negative emotions, or psychosocial difficulties (Adefope & Bayraktar, 2025; Amin et al., 2025). Although gaming may provide short-term relief, excessive engagement can reinforce avoidance behaviours, emotional dependence, and psychological distress over time (Gong et al., 2026).

Empirical evidence consistently shows that excessive gaming is positively associated with depressive symptoms, including low mood and emotional exhaustion. It is also linked to anxiety symptoms, particularly social and generalised anxiety (Amin et al., 2025; Salem, 2026c). These effects are often intensified in competitive gaming environments, where performance pressure and fear of failure are common. Such conditions are especially relevant in higher education contexts, where students experience high academic demands and major life transitions.

Excessive gaming is further associated with lower personal well-being, including reduced life satisfaction and impaired emotional balance. This relationship can be partly explained by the displacement of essential activities, such as sleep, physical activity, and offline social interaction. These patterns highlight the importance of addressing excessive gaming within preventive mental health frameworks. They also align with broader public health priorities, including Sustainable Development Goal 3, which focuses on health and well-being.

In contrast, the relationship between excessive gaming and hostile behaviours remains inconsistent. Some studies report increased aggression in competitive or violent gaming contexts (Gibson et al., 2025; Jouhki et al., 2026). However, other studies find weak or non-significant associations, particularly among university populations. This inconsistency suggests that hostile behaviours are influenced more by contextual and interpersonal factors than by gaming intensity alone.

Within youth behavioural risk frameworks, excessive gaming meets key criteria of risky behaviour. It is prevalent, associated with adverse outcomes, and may persist over time. Based on the reviewed empirical evidence, the following hypotheses are proposed: H1, H2, H3, and H4.

### 2.2. AI Cyber-Shielding and Mental Health

Recent advances in artificial intelligence have enabled the development of digital systems that support safety and well-being in online environments. In gaming contexts, AI-based tools such as automated moderation systems, toxicity detection algorithms, and adaptive feedback mechanisms are increasingly used to detect harmful interactions and manage user behaviour (Salem & Khalil, 2026). These systems operate at the interface level and are experienced through observable outcomes, including content filtering, warning prompts, and behavioural flagging.

The role of AI cyber-shielding can be explained through the technology affordance theory and perceived environmental safety frameworks. The technology affordance theory suggests that digital systems influence user behaviour independent of the level of perceived action possibilities they provide (Estupiñá Puig et al., 2024). In gaming environments, AI-enabled systems facilitate safer interactions by reducing exposure to harmful content and reinforcing behavioural norms. These affordances shape how users interpret and evaluate digital environments. Independent of their level of gaming engagement (Lukings et al., 2026).

The perceived environmental safety theory further explains how these affordances influence psychological outcomes. Individuals evaluate their environment in terms of threat, predictability, and controllability (Amoadu et al., 2025). Digital environments that exhibit visible moderation and consistent rule enforcement are more likely to be perceived as safe and manageable. This perception reduces cognitive vigilance and emotional strain, which in turn supports lower anxiety and improved well-being (Daniels et al., 2026).

By integrating these perspectives, AI cyber-shielding can be conceptualised as a perceived environmental safety affordance. It operates by shaping users’ cognitive appraisal of their interaction environment rather than by directly altering behaviour. When users encounter automated moderation, filtered content, or rapid intervention, the environment becomes more predictable and less threatening. This is associated with lower emotional arousal and improved psychological stability (Elshaer et al., 2026).

To enhance theoretical coherence, AI cyber-shielding (AI-CS) can be explained by an integrated mechanism that links technological affordances to psychological outcomes. AI-enabled systems provide observable safety affordances (e.g., content filtering, behavioural flagging, and automated moderation). This shapes users to perceive a digital game as environmentally safe (Lukings et al., 2026). This perception influences cognitive appraisal processes by reducing perceived threat, uncertainty, and exposure to harmful interactions.

As a result, lower cognitive vigilance and emotional strain are associated with reduced depression and anxiety symptoms and improved well-being. Within this framework, AI-CS operates through a perception-based environmental pathway. In which platform-level affordances influence psychological outcomes via users’ interpretations of safety conditions.

Empirical research supports the protective role of AI-enabled systems. Studies show that AI-based moderation and support tools are associated with lower levels of depressive and anxiety symptoms in digitally mediated environments. These systems may also contribute to improved well-being by enhancing perceived support, emotional regulation, and trust in digital platforms (Alexander, 2025).

However, the relationship between AI cyber-shielding and hostile behaviours remains unclear. Hostile behaviours are often shaped by social norms, personality traits, and contextual dynamics, and technological interventions alone may not be sufficient to influence such outcomes (Amoadu et al., 2025).

In this study, AI cyber-shielding is conceptualised as an independent environmental protective factor. It does not moderate the effects of excessive gaming. Instead, it represents a distinct condition within the digital environment. That is directly associated with psychological outcomes. Its influence operates through a perceived environmental safety pathway. Where reduced exposure to harmful interactions is associated with lower psychological distress and improved well-being.

Accordingly, the following hypotheses are proposed: H5, H6, H7, and H8. To enhance conceptual clarity and summarise, the theoretical foundations supporting each proposed relationship are presented in Table 2, which presents the study hypotheses along with their formal wording and key supporting references.

## 3. Research Methods

### 3.1. Research Population and Sample

In this study, online gaming refers to interactive multiplayer environments that involve real-time communication and social interaction. These include massively multiplayer online role-playing games (MMORPGs), multiplayer online battle arena (MOBA) games, first-person shooters (FPSs), and similar competitive or cooperative platforms. Mobile games were included only if they involved synchronous interaction and communication features. Offline and single-player games were excluded.

The target population comprised Generation Z youth gamers (GZYGs). Participants were students enrolled at Saudi universities who were actively engaged in online gaming. Generation Z refers to individuals who grew up in digitally mediated environments, where online interaction, peer communication, and competitive play are part of daily life (Pachi et al., 2025).

Online gaming was selected as the study context because it is a highly interactive digital environment. It involves real-time communication, visible performance, and repeated social interaction. These features shape how students interact socially and emotionally during gameplay. They also increase exposure to harassment and hostile behaviour, particularly in competitive and multiplayer settings (Salem, 2026c).

The study was conducted during the 2025–2026 academic year. Participants were eligible if they were enrolled in a Saudi university and engaged in online gaming at least 3–4 times per week. They were also required to actively use in-game communication tools, such as text or voice chat. This criterion ensured that participants were meaningfully involved in interactive gaming environments.

Participants were aged 18–22 years, which aligns with the operational definition of Generation Z youth. Their gaming patterns indicated sustained engagement, with most participants reporting frequent weekly play and active involvement in interactive gaming environments.

As some participants were minors, parental consent and student assent were obtained in accordance with institutional ethical guidelines. Data were collected using an anonymous online questionnaire distributed through official university communication channels. After screening, the final sample included 983 students of both genders and diverse national backgrounds (see Table 3a,b).

The study used a cross-sectional design and captured a single-time snapshot of excessive gaming behaviour and associated psychological outcomes. The final sample of 983 respondents exceeded recommended thresholds for Partial Least Squares Structural Equation Modelling (PLS-SEM). Following established methodological guidance, the minimum sample size in PLS-SEM may be estimated using the 10-times rule, which recommends a sample size at least ten times the largest number of structural paths directed at any endogenous construct (Sarstedt et al., 2022b). In the present model, the maximum number of incoming structural paths was two, implying a minimum requirement of 20 observations.

This threshold is highly conservative. Methodological studies further recommend substantially larger samples, often above 200 cases, to ensure stable parameter estimation and adequate statistical power in structural models (Kock & Hadaya, 2018). The sample size of 983 provided sufficient statistical power to estimate the proposed structural relationships and enhanced the robustness of the model results.

### 3.2. Data Collection and Instrument Design

Data was collected using an anonymous, self-administered online questionnaire. This method was selected to reduce social desirability bias and to address the sensitivity of reporting risky gaming behaviour, perceived AI-enabled protection, and mental health outcomes among Generation Z participants. Participation was voluntary. All respondents were informed that their responses would remain confidential and would be used exclusively for academic research.

The survey link was distributed through official university communication channels under administrative supervision. Participants received a brief explanation of the study purpose. This explanation described excessive gaming (EG) as a behavioural risk exposure and AI cyber-shielding (AI-CS) as a perceived digital protective factor. It also outlined the study’s focus on depressive symptoms (DEP), anxiety symptoms (ANX), hostile behaviours (HB), and personal well-being (PWB). Because the study involved youth participants, assent procedures were implemented in accordance with institutional ethical guidelines.

The questionnaire consisted of four structured sections. The first section collected demographic and gaming-related information, including age, gender, nationality, weekly gaming hours, and primary gaming style (competitive, cooperative, or casual). These variables were included as control variables to account for individual differences in gaming exposure.

The second section measured excessive gaming (EG) using four items (EG1–EG4) adapted from previous studies (Adefope & Bayraktar, 2025; Jouhki et al., 2026). EG was treated as the primary behavioural risk exposure in the study. The items assessed difficulty controlling gaming time, interference with academic or daily activities, and compulsive gaming tendencies. These items were adapted from validated measures of problematic gaming and Internet gaming disorder. Higher scores indicated greater levels of excessive gaming.

The third section measured mental health and well-being outcomes. Depressive symptoms (DEP) were assessed using four items (DEP1–DEP4) adapted from prior studies (Almuhtadi & Alageel, 2025; Peng et al., 2026). Anxiety symptoms (ANX) were measured using four items (ANX1–ANX4) adapted from established instruments (Ali, 2025; Pachi et al., 2025). Hostile behaviours (HB) were assessed through four items (HB1–HB4) adapted from earlier studies (Asikainen et al., 2018; Odgers & Jensen, 2020). These items captured tendencies toward irritability and antagonistic interactions in digital environments. Personal well-being (PWB) was measured using four items (PWB1–PWB4) adapted from validated well-being scales (Salem, 2026c; Smyth et al., 2010). These items reflected life satisfaction and emotional balance.

All items were measured using Likert-type scales. Higher scores indicated greater levels of depressive symptoms, anxiety symptoms, and hostile behaviours, whereas higher scores for personal well-being indicated better psychological functioning. In the structural model, excessive gaming (EG) was specified as a predictor of mental health and well-being outcomes. AI cyber-shielding (AI-CS) was modelled as an independent perceived protective factor associated with depressive symptoms, anxiety symptoms, hostile behaviours, and personal well-being. These relationships were examined using PLS-SEM.

A key methodological issue in this study concerned the measurement of AI cyber-shielding. The AI-CS items were designed to capture respondents’ perceptions of AI-enabled safety and protective features in gaming-related digital environments rather than to provide objective verification of specific platform-level technologies. This approach ensured alignment between the construct definition and the self-report method. Participants were expected to report whether they noticed, encountered, and perceived the usefulness of protective features, but they were not expected to possess technical knowledge of the underlying AI systems. Accordingly, the construct reflected users’ perceptions of enacted system interventions rather than abstract beliefs about artificial intelligence.

More specifically, the AI-CS items assessed three dimensions: perceived awareness of protective features, perceived availability of those features in the platforms used, and perceived usefulness of those features in reducing harmful digital experiences. The construct was, therefore, not intended to measure general favourability toward AI or general trust in digital technology. Instead, it focused on participants’ perceived experiences of concrete protective affordances in gaming environments. This distinction is important because it anchors the construct in platform experience rather than in broad technological attitudes.

To strengthen construct validity, the AI-CS items were explicitly anchored in observable platform-level interactions. Respondents were asked to evaluate the extent to which they notice, encounter, and benefit from AI-enabled safety mechanisms during gaming-related activity. Example item formulations included: “I notice that harmful or abusive messages are automatically filtered during gameplay or communication”, “The platforms I use provide automated warnings or actions when players behave inappropriately”, “AI-based systems help reduce harmful interactions in the gaming environments I use”, and “I experience platform features that actively monitor and manage toxic behaviour”.

These items referred to concrete, user-facing events such as content filtering, automated warnings, and behavioural flagging rather than to general beliefs about artificial intelligence. This design reduced the risk that the construct would capture abstract perceptions of safety or global attitudes toward AI. Instead, AI-CS reflected perceived experiential exposure to AI-enabled moderation and protection mechanisms, consistent with established approaches in digital environment and platform research (Frank, 2025).

A related concern in perception-based measurement is whether self-reported exposure reflects actual interaction with platform-level systems or merely general attitudes toward technology. To address this concern, the present study operationalised AI cyber-shielding in terms of observable interaction outcomes, including automated filtering of harmful messages, warning prompts, behavioural flagging, and real-time moderation responses. Prior empirical work in digital platform environments suggests that users’ perceptions of such system outputs are closely aligned with their actual exposure to moderation mechanisms, even when the underlying algorithms remain opaque (Fazeli et al., 2020; Frank, 2025). Because users directly experience the outputs of these systems through interface-level interactions, perception-based measures provide a defensible proxy for exposure in behavioural research.

The measurement of AI cyber-shielding followed established procedures for developing perception-based constructs in digital environment research. Because no validated scale currently exists to capture perceived exposure to AI-enabled safety mechanisms in gaming environments, the items were developed by adapting concepts from three related areas: perceived online safety and platform governance, the technology affordance theory, and perceived system responsiveness in digital platforms. Prior studies indicate that users’ perceptions of moderation visibility, platform intervention, and safety affordances can be measured reliably with self-report instruments when anchored in observable interaction experiences rather than technical knowledge (Seering et al., 2021; Im et al., 2022).

Item development followed a content-validity approach. Each item was explicitly linked to a user-observable outcome of AI-enabled moderation systems, such as content filtering, automated warnings, or behavioural flagging. This ensured conceptual alignment between the construct’s definition and its empirical indicators. A pilot review was conducted with subject-matter experts in digital behaviour and educational technology to assess item clarity, relevance, and representativeness. Minor wording revisions were made to improve interpretability for university students.

Construct validity was further evaluated through the measurement model. Indicator loadings met acceptable thresholds. Cronbach’s alpha and composite reliability indicated strong internal consistency. Convergent and discriminant validity criteria were also satisfied. Importantly, AI-CS demonstrated empirical distinctiveness from the psychological outcome variables. These findings imply that the construct captured perceived environmental protection rather than emotional state or general attitudes toward AI (Sarstedt et al., 2022b).

Although AI-CS was carefully anchored in observable platform-level experiences, it remains a perception-based construct and does not directly verify respondents’ exposure to specific AI systems. This limitation should be acknowledged. Future research should complement self-reported measures with behavioural or platform-level data, such as system logs or usage traces, to provide further validation.

Although AI cyber-shielding is grounded in observable platform-level mechanisms, its measurement relies on perceived exposure rather than direct system-level verification. Prior research supports the use of perception-based proxies in digital environments, particularly when system-level data are inaccessible (Magarian & Seering, 2021).

However, such measures may not fully capture actual exposure to AI-enabled moderation processes. Future research should integrate behavioural or platform-generated data (e.g., moderation logs, system-triggered interventions, or usage traces) to establish criterion validity and strengthen measurement robustness.

To further strengthen measurement validity, instrument development followed a structured content validation process. All items were explicitly mapped to theoretical constructs derived from the prior literature. An expert review was conducted to ensure conceptual clarity and relevance. The measurement model results confirmed strong reliability (Cronbach’s α and composite reliability > 0.70) and validity (AVE > 0.50; HTMT < 0.85), supporting the instrument’s robustness.

### 3.3. Common Method Variance (CMV) Concerns

Common method variance (CMV) is a recognised concern in behavioural, psychological, and educational research, especially when key constructs such as excessive gaming (EG), AI cyber-shielding (AI-CS), and mental health outcomes are measured using self-reported data collected from the same respondents at a single point in time. If not addressed, CMV may inflate observed associations and weaken the validity of structural estimates. Given the design of the present study, both procedural and statistical remedies were implemented to reduce this risk (Fuller et al., 2016). Several procedural remedies were incorporated into the survey design and administration. The wording of items was carefully designed to avoid leading, evaluative, or emotionally loaded phrasing. Different response formats were used where appropriate to reduce response patterning.

In addition, constructs were conceptually and psychologically separated across questionnaire sections. Measures of excessive gaming and AI cyber-shielding were presented before the mental health and well-being outcomes. This sequencing reduced priming effects and limited respondents’ tendency to infer causal relationships among variables. Questionnaire length was also optimised to minimise fatigue and inattentive responding (Sarstedt et al., 2022a).

Statistical remedies were then applied to evaluate CMV; (i) Harman’s single-factor test was conducted. The largest unrotated factor accounted for 41% of the total variance, which is below the 50% threshold commonly used to indicate substantial method bias. This result suggested that CMV was unlikely to be a dominant concern.

(ii) A common latent factor approach was tested using confirmatory factor analysis (CFA). The addition of a common latent factor did not meaningfully improve model fit relative to the baseline model (ΔCFI = 0.004; ΔRMSEA = 0.003). This finding provided further evidence that method bias was not substantial.

(iii) The study applied the full collinearity test using variance inflation factors (VIFs), as recommended by (Kock & Hadaya, 2018). All latent constructs showed VIF values below the conservative threshold of 3.3. This result indicated that common method bias was unlikely to confound the observed relationships. These tests provide more robust evidence in which CMV did not materially affect the model estimates.

Additional data-quality checks were performed during data collection. Automated screening procedures were used to identify straight-lining responses, extreme outliers, and duplicate IP entries. Responses with completion times below 40% of the median survey duration were removed (n = 11). No duplicate responses were retained. Enumerator-assisted submissions were also monitored using independent administrative logs to ensure adherence to standardised procedures.

Missing data was minimal, accounting for less than 2% across all items. Little’s MCAR test was not statistically significant (χ^2^ = 14.82, *p* = 0.26), supporting the assumption that the missingness was completely at random. Given the low proportion and random nature of the missingness, expectation–maximisation (EM) imputation was applied before PLS-SEM analysis. To further reduce possible order effects, questionnaire sections were counterbalanced during pilot testing, and no statistically significant differences in mean scores were observed between split forms (*p* > 0.05) (Hair et al., 2012).

### 3.4. Software, Versions, and Reproducibility Details

All analyses were conducted using the following software and settings to support reproducibility. SmartPLS 4.0 (SmartPLS GmbH, Germany) was used for the primary PLS-SEM analysis, moderation modelling, bootstrapping with 5000 subsamples, MICOM measurement invariance testing, and simple-slope visualisation. IBM SPSS Statistics 29 was used for data screening, descriptive statistics, missing-data diagnostics, and preliminary robustness checks. AMOS 29 was used to run a confirmatory factor analysis (CFA) with maximum likelihood estimation to assess standard method variance. Key analytical settings included the path weighting scheme, mean-centring of indicators for interaction terms, bias-corrected bootstrap confidence intervals, and two-tailed significance testing at α = 0.05. These details are reported to enable independent replication of the whole analytical workflow.

## 4. Results

To evaluate the hypothesised relationships, Partial Least Squares Structural Equation Modelling (PLS-SEM) was employed as the primary analytical technique. The analysis was conducted using SmartPLS version 4 (Dahri et al., 2024). A bootstrapping procedure with 5000 resamples was applied to estimate the statistical significance of structural paths, and was consistent with established methodological guidelines (Sarstedt et al., 2022b). The model evaluation was conducted in two stages: (i) The measurement model was assessed to verify indicator reliability, internal consistency, convergent validity, and discriminant validity. (ii) The structural model was evaluated to examine the hypothesised relationships among excessive gaming (EG), AI cyber-shielding (AI-CS), and psychological outcomes, while controlling demographic and gaming-related variables.

Table 4 presents the measurement model quality metrics. Indicator loadings ranged from 0.69 to 0.88, indicating acceptable indicator reliability. One item (HB4 = 0.69) was slightly below the 0.70 threshold but was retained due to its theoretical relevance and minimal association with construct reliability. Hence, the indicators adequately represent their latent constructs. Cronbach’s alpha (α) values ranged 0.84–0.90. Composite reliability (CR) values ranged 0.89–0.93. All values exceeded the recommended threshold (0.70), indicating strong internal consistency across the constructs of EG, DEP, ANX, HB, PWB, and AI-CS.

Convergent validity was also supported. Average variance extracted (AVE) values ranged from 0.61 to 0.68, exceeding the recommended cutoff of 0.50. Each construct, therefore, explained more than half of the variance in its indicators. Taken together, these results confirm that the measurement model meets the criteria for indicator reliability, internal consistency, and convergent validity. This supports the suitability of the measurement model for subsequent structural model analysis and hypothesis testing.

**Figure 1:**
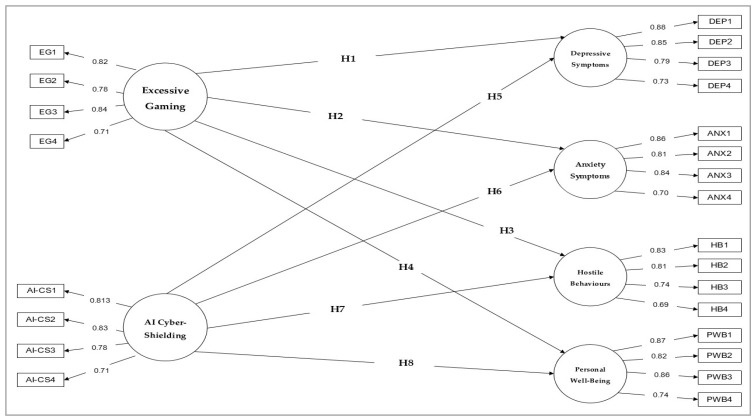
Statistical model.

Figure 1 illustrates the structural model tested in this study. The model includes two sets of direct structural relationships. Excessive gaming (EG) is specified as a behavioural risk exposure predicting depressive symptoms (DEP), anxiety symptoms (ANX), hostile behaviours (HB), and personal well-being (PWB). AI cyber-shielding (AI-CS) is defined as an independent, perceived digital protective factor associated with the same outcomes. In addition, demographic and gaming-related characteristics, including age, gender, weekly gaming hours, and gaming style, were incorporated as control variables to account for potential individual differences in gaming exposure and psychological outcomes.

Table 5 reports that excessive gaming (EG) demonstrates moderate positive correlations with depressive symptoms (r = 0.54) and anxiety symptoms (r = 0.51), and weaker negative correlations with hostile behaviours (r = −0.18) and personal well-being (r = −0.47). These associations are theoretically consistent with the excessive gaming concept as a risky behavioural exposure primarily linked to internalising mental health outcomes rather than externalised behavioural responses.

AI cyber-shield (AI-CS) exhibits moderate negative correlations with depressive symptoms (r = −0.44) and anxiety symptoms (r = −0.46), alongside a moderate positive correlation with personal well-being (r = 0.53). Significantly, none of these correlations exceed the corresponding square roots of AVE, confirming that AI cyber-shield remains empirically distinct from mental health and well-being constructs.

Hence, the Fornell–Larcker results provide clear evidence of discriminant validity among all constructs—excessive gaming, depressive symptoms, anxiety symptoms, hostile behaviours, personal well-being, and AI cyber-shield.

**Figure 2:**
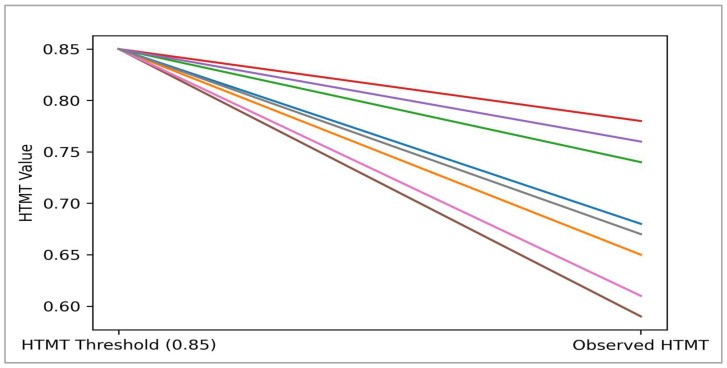
HTMT matrix.

Figure 2 presents the highest HTMT value, which is observed between depressive symptoms and personal well-being (HTMT = 0.78), followed by the association between anxiety symptoms and personal well-being (HTMT = 0.76).

While these values reflect strong conceptual relatedness, such associations are theoretically expected given the close linkage between associated with lower psychological distress and subjective well-being. Significantly, none of the values approaches the critical threshold, suggesting that the constructs remain empirically distinct.

The HTMT values involving AI cyber-shield and the mental health constructs are comparatively lower (AI-CS–DEP = 0.59; AI-CS–ANX = 0.61; AI-CS–PWB = 0.67), indicating that AI cyber-shield represents a conceptually distinct preventive construct rather than a proxy for psychological symptomatology or well-being.

**Figure 3:**
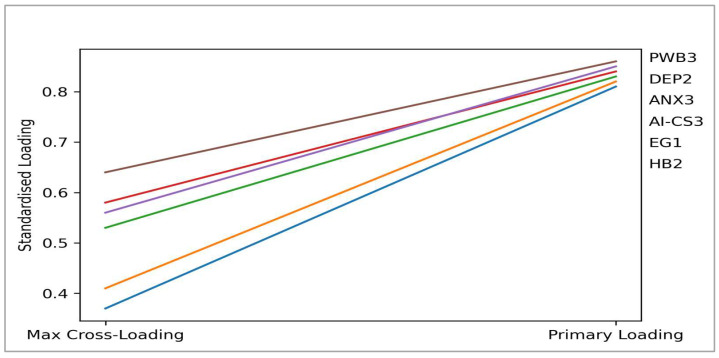
Indicator loadings and cross-loadings.

Figure 3 reports that EG1 exhibits a strong loading on excessive gaming (0.82) and substantially lower loadings on depressive symptoms (0.41), anxiety symptoms (0.39), hostile behaviours (−0.12), personal well-being (−0.36), and AI cyber-shield (−0.18). Similarly, DEP2 and ANX3 load most strongly on depressive symptoms (0.85) and anxiety symptoms (0.84), respectively, with clearly weaker cross-loadings on non-target constructs.

Indicators representing hostile behaviours (HB2), personal well-being (PWB3), and AI cyber-shield (AI-CS3) demonstrate the expected pattern, with high primary loadings (0.81, 0.86, and 0.83, respectively) and comparatively lower cross-loadings across the remaining constructs. Although some cross-loadings between mental health indicators and personal well-being are moderate, these associations are theoretically meaningful and remain well below the indicators’ primary loadings.

Thus, the cross-loading results confirm that all indicators are empirically aligned with their intended constructs, thereby supporting indicator reliability and discriminant validity. These findings are consistent with evidence from the Fornell–Larcker criterion and the HTMT analysis, further supporting the adequacy of the measurement model.

All structural relationships were estimated while controlling demographic and gaming-related variables. The inclusion of these control variables helps ensure that the observed associations between excessive gaming, AI cyber-shielding, and psychological outcomes are not confounded by differences in age, gender, gaming intensity, or gaming style among participants.

As seen in Table 6, excessive gaming (EG) exhibits a significant positive association with depressive symptoms (β = 0.46, t = 14.21, *p* < 0.001) and anxiety symptoms (β = 0.43, t = 13.07, *p* < 0.001), thereby supporting (H1–H2). These findings indicate that excessive gaming functions as a salient risky behavioural exposure associated with increased internalising mental health symptoms among Gen Z youth.

Table 6 presents, the path from excessive gaming to hostile behaviours is negative but not statistically significant (β = −0.06, t = 1.42, *p* = 0.155), thereby failing to support H3. This suggests that excessive gaming does not directly translate into hostile or antagonistic behaviours in the sampled population. However, excessive gaming shows a significant negative association with personal well-being (β = −0.39, t = 11.84, *p* < 0.001), supporting H4 and indicating that higher levels of gaming exposure are linked to lower subjective well-being.

Regarding the preventive role of AI cyber-shield (AI-CS), the results reveal significant negative associations with both depressive symptoms (β = −0.32, t = 9.67, *p* < 0.001) and anxiety symptoms (β = −0.35, t = 10.21, *p* < 0.001), supporting H5 and H6. These findings indicate that perceived AI-based cyber-shielding is associated with lower depression and anxiety symptoms among youth gamers.

The direct association between AI cyber-shield and hostile behaviours is negative but not statistically significant (β = −0.05, t = 1.18, *p* = 0.239), thereby failing to support H7. This indicates that AI cyber-shielding is not directly associated with hostile behavioural tendencies. In contrast, AI cyber-shield shows a strong, significant positive association with personal well-being (β = 0.48, t = 15.03, *p* < 0.001), thereby supporting H8 and underscoring its link to higher overall well-being.

Hence, the structural model results demonstrate a clear pattern in which excessive gaming primarily affects mental health and well-being outcomes, rather than hostile behaviours. At the same time, AI cyber-shielding is associated with psychological outcomes in a direction consistent with a protective environmental factor for personal well-being. The non-significant paths involving hostile behaviours suggest that such behaviours may be associated with factors beyond gaming exposure and AI-based protection, warranting further investigation.

**Figure 4:**
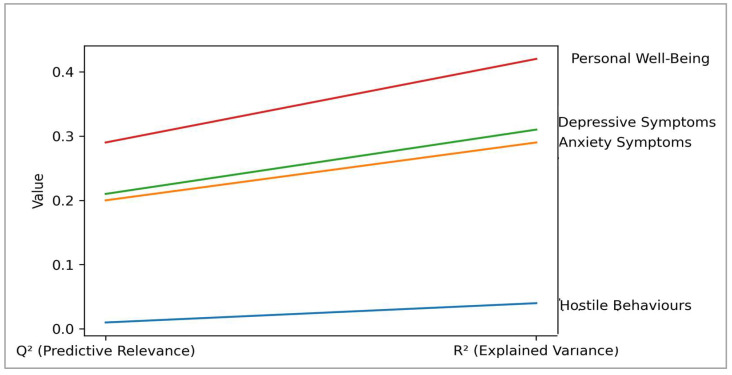
Structural model quality metrics.

Figure 4 shows that the model has moderate to strong explanatory power for mental health and well-being outcomes. It explains 31% of the variance in depressive symptoms (R^2^ = 0.31) and 29% of the variance in anxiety symptoms (R^2^ = 0.29). These values are considered moderate in behavioural and mental health research. The model explains a higher proportion of variance in personal well-being (R^2^ = 0.42), indicating strong explanatory power for this outcome among Gen Z gamers.

In contrast, the explained variance for hostile behaviours is low (R^2^ = 0.04). This suggests that excessive gaming and AI cyber-shielding have a limited direct association with hostile behaviours. This result is consistent with the non-significant structural paths and indicates that other factors may better explain this outcome.

Association strength results further clarify these relationships. Excessive gaming shows moderate to significant associations with depressive symptoms (f^2^ = 0.27) and anxiety symptoms (f^2^ = 0.24). It also shows a strong association with personal well-being (f^2^ = 0.31). AI cyber-shielding demonstrates moderate associations with depressive symptoms (f^2^ = 0.18) and anxiety symptoms (f^2^ = 0.22), and a strong association with personal well-being (f^2^ = 0.38). These findings highlight the meaningful role of both predictors in shaping mental health and well-being.

By contrast, association strength for hostile behaviours is negligible (f^2^ ≤ 0.02). This further confirms that the model does not adequately explain hostile behaviours. All endogenous constructs show positive predictive relevance (Q^2^ > 0). Predictive relevance is moderate for depressive symptoms (Q^2^ = 0.21), anxiety symptoms (Q^2^ = 0.20), and personal well-being (Q^2^ = 0.29). The predictive relevance of hostile behaviours is minimal (Q^2^ = 0.01).

Thus, the model demonstrates adequate explanatory and predictive power for mental health and well-being outcomes among Gen Z gamers. The findings confirm excessive gaming as a significant mental health risk and highlight AI cyber-shielding as a meaningful protective correlation within gaming-related digital environments. However, hostile behaviours appear to be associated with additional social, contextual, or personality-related factors that require further investigation.

## 5. Discussion

This study examined an association-based model in which excessive gaming (EG) was conceptualised as behavioural risk exposure, and AI cyber-shielding (AI-CS) was conceptualised as a perceived digital protective condition among Generation Z university students. The model assessed how these constructs relate to four psychological outcomes: depressive symptoms, anxiety symptoms, hostile behaviours, and personal well-being.

The findings show that EG and AI-CS operate as independent dimensions rather than interacting factors. EG reflects behavioural exposure, whereas AI-CS reflects perceived environmental conditions. Importantly, the results indicate statistical associations rather than causal effects due to the cross-sectional design. Therefore, the findings should be interpreted as patterns of co-occurrence rather than evidence of causal influence. Longitudinal and experimental studies are required to establish causal mechanisms (Alexander, 2025; Lukings et al., 2026).

Excessive gaming was positively associated with depressive and anxiety symptoms and negatively associated with personal well-being. This pattern indicates that higher levels of gaming dysregulation are linked to increased psychological distress and reduced overall functioning. One plausible explanation is that excessive gaming may act as a short-term coping strategy while co-occurring with longer-term emotional strain. It may also displace restorative activities such as sleep, physical activity, and offline social interaction. These interpretations are consistent with prior research linking intensive digital engagement to lower well-being and higher distress (Salem, 2026b).

In contrast, excessive gaming was not significantly associated with hostile behaviours. These findings imply that gaming intensity alone does not explain externalised behavioural outcomes. Hostile behaviour in digital environments appears to depend more on contextual and social factors. These include competitive dynamics, peer norms, and exposure to toxic communication (Zhang et al., 2025). This distinction indicates that internal psychological outcomes and external behaviours follow different explanatory pathways.

AI cyber-shielding showed the opposite pattern. It was negatively associated with depressive and anxiety symptoms and positively associated with personal well-being. This indicates that perceived exposure to AI-enabled safety mechanisms is linked to lower distress and better psychological functioning. AI-CS, as conceptualised in this study, reflects perceived environmental safety rather than behavioural control. It captures user perceptions of moderation systems, content filtering, and adaptive platform responses.

However, AI-CS was not significantly associated with hostile behaviours. This suggests that technological protection alone is insufficient to influence outward behavioural expressions. Hostile behaviour likely requires additional social, behavioural, and institutional interventions beyond platform-level mechanisms (Amin et al., 2025).

Taken together, the findings support a clear interpretation. Excessive gaming is associated with higher distress and lower well-being, while AI cyber-shielding is associated with lower distress and higher well-being. These constructs represent two distinct but complementary dimensions: behavioural exposure and perceived environmental protection. Their independent effects highlight the importance of analysing risk and protection separately within digital environments.

From a theoretical perspective, the study extends existing research by shifting the focus from behaviour-centric models to environment-inclusive models of digital well-being. The results indicate that psychological outcomes are associated not only with user behaviour but also with perceived platform conditions, such as safety, predictability, and responsiveness. This aligns with prior work emphasising the role of environmental safety in shaping emotional outcomes (Odgers & Jensen, 2020).

Within this framework, AI cyber-shielding can be conceptualised as a perceived safety affordance. Unlike traditional protective factors, it reflects algorithmically mediated environmental regulation. AI systems actively shape user experience through moderation, filtering, and adaptive feedback. This positions digital platforms as active agents that influence psychological outcomes rather than as passive environments (Tateno et al., 2025; Zhang et al., 2025).

From a practical perspective, the findings suggest that addressing gaming-related risks requires a multi-level approach. Reducing excessive gaming may help mitigate psychological distress. At the same time, strengthening AI-enabled safety mechanisms may enhance perceived psychological safety. However, the absence of effects on hostile behaviour indicates that technological solutions alone are insufficient. Effective intervention should integrate technological, social, and educational strategies.

In summary, the study demonstrates that excessive gaming and AI cyber-shielding are independently associated with psychological outcomes in opposite directions. These findings provide a basis for future research, particularly longitudinal and multi-method studies to examine causal pathways and interaction effects (Amin et al., 2025; Pachi et al., 2025; Salem, 2026a).

Finally, because all variables were measured using self-reported data from a single survey, common method variance (CMV) was considered. Procedural remedies were applied, including anonymity and neutral wording. Harman’s single-factor test indicated that no single factor accounted for the majority of variance. In addition, full collinearity assessment showed that all variance inflation factors (VIFs) were below 3.3 (Fuller et al., 2016; Kock & Hadaya, 2018). These results suggest that CMV is unlikely to significantly bias the findings. However, CMV cannot be fully excluded in cross-sectional designs. Future studies should use multi-source and longitudinal data to strengthen validity.

## 6. Theoretical and Practical Contributions

The primary contribution of this study is conceptual rather than empirical. The statistical associations are broadly consistent with the existing literature on excessive gaming and mental health. However, the study advances the field by introducing a clearly defined, measurable construct of AI-enabled protection in digital environments. Specifically, the study conceptualises and operationalises AI cyber-shielding (AI-CS) as a form of perceived environmental protection. This construct is distinct from both behavioural exposure and general attitudes toward artificial intelligence. Previous research has examined excessive gaming as a behavioural risk factor and has separately analysed AI-based moderation systems as technical features of digital platforms. However, these research streams have remained largely disconnected. AI-enabled protection has not been translated into a construct that can be empirically integrated into behavioural models of mental health and well-being.

By defining AI cyber-shielding as users’ perceived exposure to AI-enabled safety mechanisms, the study provides an operational framework grounded in observable platform interactions. These mechanisms include moderation systems, content filtering, and behavioural flagging. This approach shifts the analytical focus from AI as background infrastructure to AI as a user-experienced environmental condition with psychological relevance.

As a result, platform-level protection becomes measurable within behavioural research. The study further contributes by advancing a dual-condition model of digital well-being. In this model, psychological outcomes are associated with both behavioural risk exposure (excessive gaming) and perceived environmental protection (AI cyber-shielding). This framework extends beyond behaviour-centric models by incorporating environmental conditions as independent determinants of psychological functioning.

This distinction has theoretical implications. It demonstrates that digital well-being cannot be explained solely by user behaviour. Instead, it is also associated with individuals’ perceptions of the safety, predictability, and responsiveness of their digital environment. These factors operate in parallel with behavioural exposure rather than as statistical interaction effects.

In this way, this study extends the Problem Behaviour Theory into digitally mediated contexts by incorporating platform-level protective affordances as independent explanatory factors. The contribution of the study lies in refining the conceptualisation and measurement of protection in digital environments. It establishes a foundation for integrating AI-enabled environmental conditions into future models of youth mental health and digital well-being.

The practical implications are direct. Universities can use these findings to identify students at risk of excessive gaming and to implement targeted prevention strategies. These strategies may include programmes focused on emotional regulation, digital balance, and mental health awareness. Counselling services can incorporate brief screening tools to assess gaming-related risk.

AI-enabled safety mechanisms can also be integrated into digital platforms used by students. These include automated moderation systems, behavioural alerts, adaptive safety prompts, and referral features. Such tools offer scalable support, particularly in contexts where mental health resources are limited.

The study advances theoretical understanding by demonstrating that perceived environmental conditions within digital platforms function as independent determinants of psychological outcomes, rather than merely contextual background factors. This shifts the analytical focus from behaviour-centric models that dominate gaming and digital well-being research toward an environment–behaviour dual-condition framework. Within this framework, user behaviour (e.g., excessive gaming) and perceived platform characteristics (e.g., AI-enabled safety affordances) operate as distinct but complementary influences on mental health.

Importantly, this reconceptualisation extends existing models of digital well-being by introducing perceived platform-level protection as a measurable psychological determinant, thereby bridging the gap between technical system design and user-level psychological outcomes. This contribution provides a foundation for future research to integrate platform affordances into behavioural models, advancing both theoretical precision and interdisciplinary integration between digital technology research and mental health research.

## 7. Conclusions

This study examined excessive gaming (EG) as a behavioural risk exposure and AI cyber-shielding (AI-CS) as a perceived digital protective factor among Generation Z university students. The findings provide consistent evidence that excessive gaming is associated with adverse psychological outcomes. Specifically, excessive gaming showed a positive association with depressive symptoms and anxiety symptoms, and a negative association with personal well-being. However, it was not significantly associated with hostile behaviours.

AI cyber-shielding demonstrated an opposite pattern of associations. It was negatively associated with depressive and anxiety symptoms and positively associated with personal well-being. No significant relationship was observed between AI cyber-shielding and hostile behaviours. These results indicate that both excessive gaming and AI cyber-shielding are primarily associated with internal psychological outcomes rather than externalised behavioural responses.

The findings support a conceptual distinction between behavioural risk exposure and perceived environmental protection in digital contexts. Excessive gaming reflects a pattern of dysregulated engagement that is associated with emotional distress and reduced well-being. In contrast, AI cyber-shielding reflects users’ perceived exposure to AI-enabled safety mechanisms embedded in gaming-related digital environments. These mechanisms include content moderation, toxicity detection, behavioural flagging, and automated intervention features that are observable at the user interface level.

Importantly, AI cyber-shielding does not represent a single standardised technological system. It captures users’ perceived experience of protective affordances across multiple platforms and interaction environments. This perception-based operationalisation is appropriate in a self-reported design, where respondents report experienced system effects rather than underlying technical architectures. Accordingly, the construct reflects experiential exposure to platform-level safety mechanisms rather than abstract attitudes toward artificial intelligence.

The absence of significant associations with hostile behaviours suggests that externalised outcomes may follow different explanatory pathways. Such behaviours are likely shaped by broader social, contextual, and individual factors that extend beyond gaming intensity and platform-level protection.

Moreover, the study contributes to the literature by distinguishing between behavioural and environmental determinants of digital well-being. It demonstrates that psychological outcomes in gaming contexts are associated not only with user behaviour but also with users’ perceptions of how safe and regulated their digital environment is. This distinction provides a more precise basis for understanding mental health in digitally mediated environments and highlights the relevance of AI-enabled protective systems as part of broader preventive strategies in higher education contexts.

## 8. Limitations and Future Research

This study has several limitations that should be considered when interpreting the findings. First, the cross-sectional design does not allow for causal inference. The observed relationships reflect statistical associations rather than directional effects. Future research should employ longitudinal or experimental designs to establish temporal ordering and causal mechanisms. Second, all variables were measured using self-reported data collected within a single survey. This approach may introduce common method bias and social desirability effects, despite the procedural and statistical controls applied. Future studies should incorporate multi-method approaches, including behavioural data, platform-generated logs, or clinical assessments, to strengthen measurement validity.

Third, the model shows limited explanatory power for hostile behaviours. This suggests that such outcomes are influenced by factors not captured in the present framework. Variables such as personality traits, peer norms, competitive dynamics, and offline stressors may play a more central role. Future research should extend the model to include these factors and examine alternative explanatory pathways. Fourth, AI cyber-shielding was operationalised as a single higher-order construct that reflects perceived exposure to AI-enabled safety mechanisms. This approach supports conceptual clarity but may obscure differences between specific types of AI interventions. Future studies should disaggregate this construct and examine distinct components, such as content moderation, behavioural flagging, and adaptive feedback systems, to identify their differential effects.

Finally, the sample was limited to Generation Z university students within a specific regional context. This may constrain the generalisability of the findings. Future research should test the proposed model across diverse age groups, cultural settings, and gaming environments to assess its robustness and external validity.

## Figures and Tables

**Table 1:** Research objectives and questions.

Research Objective	Research Question
RO1. To examine whether EG is associated with DEP among Gen Z youth	RQ1. Is EG associated with DEP among Gen Z youth?
RO2. To examine whether EG is associated with ANX among Gen Z youth	RQ2. Is EG associated with ANX among Gen Z youth?
RO3. To examine whether EG is associated with HB among Gen Z youth	RQ3. Is EG associated with HB among Gen Z youth?
RO4. To examine whether EG is associated with PWB among Gen Z youth	RQ4. Is EG associated with PWB among Gen Z youth?
RO5. To examine whether AI-CS is associated with DEP	RQ5. Is AI-CS associated with DEP among Gen Z youth?
RO6. To examine whether AI-CS is associated with ANX	RQ6. Is AI-CS associated with ANX among Gen Z youth?
RO7. To examine whether AI-CS is associated with HB	RQ7. Is AI-CS associated with HB among Gen Z youth?
RO8. To examine whether AI-CS is associated with PWB	RQ8. Is AI-CS associated with PWB among Gen Z youth?

**Table 2:** Hypotheses and supporting literature.

RH	Statement	Supporting References
RH1	Excessive gaming (EG) is positively associated with depressive symptoms (DEP).	(Pachi et al., 2025)
RH2	Excessive gaming (EG) is positively associated with anxiety symptoms (ANX).	(Amin et al., 2025; Pachi et al., 2025; Salem, 2026a)
RH3	Excessive gaming (EG) is associated with hostile behaviours (HB).	(Phillips, 2025)
RH4	Excessive gaming (EG) is negatively associated with personal well-being (PWB).	(Almuhtadi & Alageel, 2025)
RH5	AI cyber-shielding (AI-CS) is negatively associated with depressive symptoms (DEP).	(Tateno et al., 2025; Zhang et al., 2025)
RH6	AI cyber-shielding (AI-CS) is negatively associated with anxiety symptoms (ANX).	(Amin et al., 2025; Zhang et al., 2025)
RH7	AI cyber-shielding (AI-CS) is negatively associated with hostile behaviours (HB).	(Almuhtadi & Alageel, 2025)
RH8	AI cyber-shielding (AI-CS) is positively associated with personal well-being (PWB).	(Amin et al., 2025; Pachi et al., 2025; Salem, 2026b)

**Table 3:** (**a**) Demographic characteristics (N = 983). (**b**) Demographic and gaming characteristics (N = 983).

(**a**)						
**Nationality**	**Male**		**Female**		**N**	**%**
	**N**	**%**	**N**	**%**		
Saudi Arabia	101	10.27	85	8.65	186	18.92
Syria	84	8.55	71	7.22	155	15.77
Yemen	79	8.04	63	6.41	142	14.45
Egypt	98	9.97	99	10.07	197	20.04
Sudan	92	9.36	78	7.93	170	17.29
Jordan	79	8.04	54	5.49	133	13.53
Total	533	54.22	450	45.78	983	100
(**b**)						
**Variable**	**Category**				**N**	**%**
**Age**	18–19 years				379	38.6
	20–21 years				328	33.4
	22 years				276	28.1
**Weekly gaming hours**	<15 h				81	8.2
	15–30 h				389	39.6
	>30 h				513	52.2
**Primary game played** *	Roblox (Roblox Corporation)				189	19.2
	Minecraft (Mojang Studios)				407	41.4
	Fortnite (Epic Games)				300	30.5
	Valorant (Riot Games)				601	61.1
	League of Legends (Riot Games)				210	21.4
	Call of Duty (Activision)				503	51.2
	PUBG (PUBG Corporation)				805	81.9
	Genshin Impact (miHoYo)				871	88.6
	Other games				98	9.9

* Participants could report more than one game; therefore, frequencies may exceed the total sample size.

**Table 4:** Measurement model quality metrics (latent constructs and indicators).

Construct	Loading	α	CR	AVE
Excessive Gaming (EG)		0.86	0.9	0.64
EG1	0.82			
EG2	0.78			
EG3	0.84			
EG4	0.71			
Depressive Symptoms (DEP)		0.89	0.92	0.66
DEP1	0.88			
DEP2	0.85			
DEP3	0.79			
DEP4	0.73			
Anxiety Symptoms (ANX)		0.88	0.91	0.65
ANX1	0.86			
ANX2	0.81			
ANX3	0.84			
ANX4	0.72			
Hostile Behaviours (HB)		0.84	0.89	0.61
HB1	0.83			
HB2	0.81			
HB3	0.74			
HB4	0.69			
Personal Well-Being (PWB)		0.9	0.93	0.68
PWB1	0.87			
PWB2	0.82			
PWB3	0.86			
PWB4	0.74			
AI Cyber-Shield (AI-CS)		0.87	0.91	0.63
AI-CS1	0.85			
AI-CS2	0.83			
AI-CS3	0.78			
AI-CS4	0.71			

Note: The items EG1–EG4, DEP1–DEP4, ANX1–ANX4, HB1–HB4, PWB1–PWB4, and AI-CS1–AI-CS4 represent observed indicators, while EG, DEP, ANX, HB, PWB, and AI-CS represent latent constructs.

**Table 5:** Fornell–Larcker criterion matrix.

Construct	EG	DEP	ANX	HB	PWB	AI-CS
EG	0.80					
DEP	0.54	0.81				
ANX	0.51	0.63	0.81			
HB	−0.18	−0.31	−0.29	0.78		
PWB	−0.47	−0.66	−0.62	0.41	0.82	
AI-CS	−0.21	−0.44	−0.46	0.19	0.53	0.79

**Table 6:** Hypotheses evaluation.

Hypothesis	Path	β	t	*p*-Value	Evaluation
H1	EG → DEP	0.46	14.21	<0.001	Accepted
H2	EG → ANX	0.43	13.07	<0.001	Accepted
H3	EG → HB	−0.06	1.42	0.155	Rejected
H4	EG → PWB	−0.39	11.84	<0.001	Accepted
H5	AI-CS → DEP	−0.32	9.67	<0.001	Accepted
H6	AI-CS → ANX	−0.35	10.21	<0.001	Accepted
H7	AI-CS → HB	−0.05	1.18	0.239	Rejected
H8	AI-CS → PWB	0.48	15.03	<0.001	Accepted

## Data Availability

The data supporting the findings of this study are available from the corresponding author upon reasonable request, subject to privacy and ethical restrictions.
